# Transgenerational telomere erosion in the monogametic sex: human telomeres progressively erode in the female germline and do not lengthen in aged testes

**DOI:** 10.1186/s13039-019-0450-4

**Published:** 2019-08-23

**Authors:** Reinhard Stindl

**Affiliations:** Alpharm GesmbH, apo-med-center, Plättenstrasse 7-9, 2380 Perchtoldsdorf, Austria

**Keywords:** Telomere erosion, Birth-cohort effect, Paternal age at conception, Transgenerational, Telomeres, Germline

## Abstract

Long telomeres, the protective caps of eukaryotic chromosomes, which erode during aging, have been the symbol of youth and regenerative potential. It therefore came as a surprise, when several cross-sectional studies reported that telomeres in sperm cells of old men are longer than in young men and that paternal age is positively linked to telomere length of children. To explain the puzzling data, several theories have been put forward, from Darwinian selection to high telomerase activity or alternative telomere lengthening in sperms of geriatrics. Unfortunately, the idea of a birth-cohort effect has been ignored, despite existing theoretical models and despite findings of progressive telomere erosion between human generations. The old theoretical model of progressive telomere erosion in the female germline is discussed here and updated with the hypothesis that progressive telomere erosion is tied to the monogametic sex in all higher animals. Longitudinal studies of germline telomere length in humans are much needed, since a limited regenerative capacity of somatic tissues will most likely result in an increase in and earlier onset of the so-called age-associated diseases.

## Introduction

For decades, long telomeres, the protective caps of eukaryotic chromosomes, have been the symbol of youth and regenerative potential in higher animals, including humans. Although current methods of telomere length measurement are error-prone, and despite frequent sampling of human blood, which is a poor choice because of its dynamical nature [1], there is strong converging evidence that telomeres erode during aging.

Lifelong tissue regeneration, which is based on ongoing tissue stem cell divisions, results in replicative telomere erosion in the somatic tissues of most higher animals. This is in contrast to the germline, where high expression levels of the enzyme telomerase have been thought to stabilize telomere lengths [2]. However, it has been known that telomere length differs widely between human populations, human individuals, human tissues, tissue cells, chromosomes of a cell, even between homologous chromosomes and arms of a chromosome [3–5]. Despite the differences, common telomere profiles in humans have been found, characterized by similar relative telomere lengths on specific chromosome arms of different individuals of a population [6]. Furthermore, monozygotic twins have similar telomere length profiles at old age [7]. The most plausible scenario is that the relative telomere lengths of specific chromosomes are inherited, because the chromosomes themselves or identical copies of them end up in the fertilized egg. With this in mind, it appears obvious that telomere lengths cannot be stable over generations, because the observed highly diverse telomere length patterns [3, 4] are rather incompatible with any embryonic leveling mechanism and instead point to an underlying transgenerational process of a dynamic nature [8, 9].

In 2005, it came as a surprise when Unryn and colleagues reported that telomeres in sperm cells of old men are longer than in young men and that paternal age is positively linked to telomere length of children [10]. To be precise, Allsopp et al. found longer telomeres in the testes of old men already in 1992 [11], but no follow-up was undertaken for more than a decade, until the confirmation of the initial findings by Unryn and colleagues.

These stunning results have been replicated by several other research groups, but no longitudinal human studies have yet been performed. Despite the warning of Unryn et al. about the possibility of a birth-cohort effect in the discussion section of their initial paper, many colleagues in the telomere field jumped to conclusions that were not supported by all the cross-sectional studies. Accordingly, the currently accepted theories can be summarized as follows: a) telomeres lengthen in the testes of seniors (by telomerase and/or alternative lengthening mechanisms); b) positive selection for sperm cells with extra-long telomeres in very old men; c) positive selection for seniors with long-telomered sperms; and d) negative selection against seniors with short telomeres in their sperms.

Among the long-time supporters of the idea that sperm telomere length increases with age in humans are Eisenberg and Kuzawa [12]. On May 29th 2019, Eisenberg, Kuzawa and colleagues presented a reanalysis of a cross-sectional multigenerational study [13] in the *Proceedings of the Royal Society B*, where they again investigated the paternal-age-at-conception effect. In line with their previous findings [12], they confirmed that older fathers produced long-telomered offspring and that a similar effect regarding the age of the grandfather (at conception of the father) exists. What they (again) showed was that an advanced age of the father had a positive effect on the telomere length of the offspring’s white blood cells, and this effect persisted over at least two generations.

If single chromosomes with specific telomere lengths are directly inherited, it is easy to understand why long-telomered chromosomes end up in the offspring. But why should geriatric males, usually characterized by extensive wear and tear in somatic tissues, harbor the fountain of youth in their testes, outcompeting every youngster’s sperm machinery by far? And how is this compatible with consistent age-dependent declines in semen quality found in numerous human studies [14], and the biological fact that nothing gets better with (advanced) age? Could it be that it is not a process of telomere lengthening or Darwinian selection within the lifetime of an individual, but, on the contrary, a simple birth-cohort effect? What if older males already had longer telomeres in their sperm cells than their younger colleagues from the start, when they were young?

## Is it a birth-cohort effect?

**Fig. 1:**
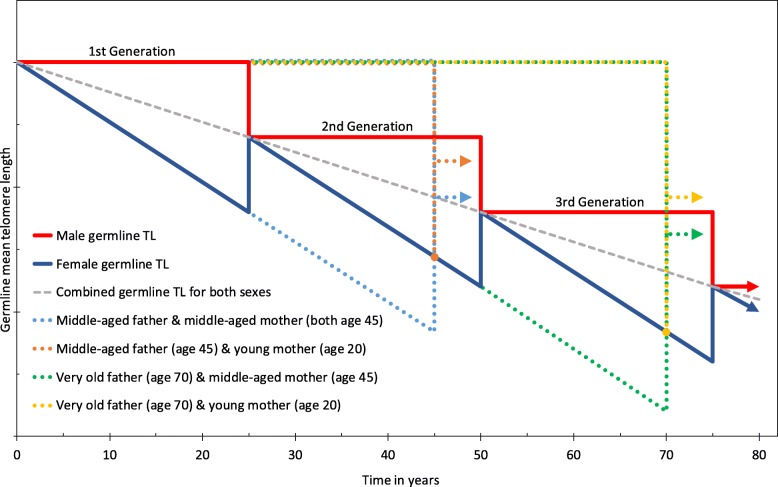
Concept of transgenerational telomere erosion in the female germline (as published in [9, 15]): High expression levels of telomerase stabilize sperm telomeres in human males throughout reproductive life (flat red line). Already at prenatal age, the ovaries are populated with millions of primary oocytes during oocytogenesis. Only a small fraction of them complete meiosis and start ovulating in a serial manner at menarche. It has been postulated that long-telomered ova are utilized first [16], whereas female germ cells with critically short telomeres are last (descending blue line). Support for this hypothesis comes from the well-known fact that middle-aged women have an increased risk of aneuploid pregnancies (e.g. Down’s syndrome), which rapidly increases during the second half of the female reproductive period [17]. In diploid organisms, at conception, one chromosome comes from the mother the other from the father, resulting in a combination of parental chromosomes and thereby telomere lengths in the fertilized egg (junctions of red & blue lines). Older males bypass the telomere loss of 1–2 generations and on average have longer sperm telomeres than their younger contemporaries. Several scenarios and combinations are highlighted in the graph (colored broken lines). The negative maternal age effect on offspring’s telomeres is masked by the fact that older women tend to have male partners of similar advanced age (blue broken line). The positive paternal age effect is pronounced, because the reproductive period of males is twice as long as in females and therefore old fathers can bypass the telomere loss of two generations (yellow and green broken lines)

In summer 2001, at the University of California at Berkeley, I started to develop a biological framework of an intrinsic mechanism of species extinction, which is based on telomere erosion in the germline of a species. “Is telomere erosion a mechanism of species extinction?” was published in 2004 [8]. Seven years later, mostly based on various signs of short-telomered ova in aged mothers (e.g. Down’s syndrome), I proposed that the source of transgenerational human telomere erosion is located in the female germ line. I further argued that the paternal-age-at-conception effect is a birth-cohort effect due to old males bypassing the female-based intergenerational telomere loss [9, 15]. Because the initial paper was published in 2011 in a peer-reviewed journal that is not listed in Medline, I republished the concept as part of an advanced evolutionary theory in *Naturwissenschaften* in 2014 [9]. Both papers contained a graph, which is shown in Fig. 1 in a slightly updated version, with special emphasis on the recent results of Eisenberg and colleagues (Fig. 1).

In 2015, in the prestigious journal *Aging Cell*, Holohan et al. reported a downward secular trend in telomere length at birth in healthy human populations in all the birth-cohorts they looked at, back to 1920 [18], as predicted [8, 9, 15]. Holohan and colleagues found clear evidence that newborns of previous generations must have had longer telomeres, but did not present a thorough survey of the existing literature [19]. They speculated that intergenerational telomere erosion could be the consequence of an environmental factor. It must be a recent phenomenon, they wrote, possibly because the implications of an evolutionary mechanism of transgenerational telomere erosion in the human lineage [9] would have been unthinkable. It has been thought for decades that human telomeres are lengthened during an early stage of embryonic development [2], yet it has been shown in numerous studies that telomere length in parental germ cells impacts telomere length in offspring cells [10–12]. It is therefore clear that the hypothetical embryonic reset to species-specific telomere lengths [16] cannot exist.

The alternative concept of why older paternal ages and grandpaternal ages at conception predict longer telomeres in human descendants is shown in Fig. 1, and it has nothing to do with telomere lengthening in sperms or Darwinian selection, as suggested by Eisenberg and colleagues [13]; to the contrary, it is about telomere erosion in the female germline and the lack of Darwinian selection against it [8, 9, 15]. According to this model, telomeres in the testes of elderly males are longer than those in contemporary young males, because seniors are members of a previous generation and therefore skipped, on average, one female-based intergenerational telomere loss (Fig. 1).

## Is there progressive telomere erosion in the female germline?

Eukaryotic cells that contain abnormal sets of chromosomes are called aneuploid. Aneuploidy is characterized by numerical variations from the normal set of chromosomes, but also (in the modern literature) by unbalanced structural aberrations of chromosomes [20]. Critically short telomeres are a well-known source of telomere associations/fusions, which subsequently result in numerical and/or structural chromosomal aberrations in dividing eukaryotic cells due to anaphase bridges [21]. By far the highest aneuploidy rate of all human cell types during aging, with the exception of cancerous cells [20], has been found in oocytes [22]. Actually, the majority of oocytes of women aged 40 years and older may be aneuploid [17]. Keefe and colleagues introduced the telomere theory of reproductive senescence in women as early as 2005. Accordingly, oocytes with long telomeres ovulate first at young age, whereas oocytes from older women have shorter telomeres due to a later exit from the production line during fetal oogenesis. However, Keefe adhered to the concept of an embryonic reset, which ensures a stable telomere length of the human lineage [16], opposing the idea of transgenerational telomere erosion [8].

In contrast, I regard the strong positive correlation between the mother’s age at conception and trisomic pregnancies [22], like Down’s syndrome, as a sign of (transgenerational) telomere erosion in the female germline [9, 15]. Despite lifelong germ cell divisions, the father being of an advanced age does not increase the incidence of chromosomally abnormal offspring [22], which further supports the concept of telomere erosion in the female germline. Of course, the relatively high rate of intergenerational telomere loss found in humans [18] suggests that it either represents an advanced stage of a fundamental natural mechanism [9], which might be exacerbated by the increasing age of mothers in civilized societies [23], or is caused by a yet unknown environmental factor [18]. An environmental factor, like industrial pollution [18], as a cause for the erosion seems to be rather unlikely, because Holohan and colleagues found a continuous telomere loss in each of the studies, spanning all generations. I cannot imagine any environmental factor that has been introduced many decades ago and has not changed since. Therefore, I am convinced that it is no environmental or extrinsic factor but an intrinsic factor on a universal scale [8, 9, 15].

## Is progressive telomere erosion in the germline tied to the monogametic sex?

Different results have been reported regarding the paternal-age-at-conception effect (PAC-effect) in various species [24]. Based on the theory of transgenerational telomere erosion [8], a positive age-at-conception effect in one sex points to transgenerational telomere erosion in the opposite sex. Whereas in chimpanzees a positive PAC-effect has been found, in birds a negative PAC-effect seems to exist. In a review on this topic, four bird species out of six are listed with a negative PAC in table 1 [24] and the two studies with a positive PAC-effect have a very high *p*-value. If this exception from the rule turns out to be true, it might be promising to investigate a possible link between progressive telomere erosion and the monogametic sex. In birds, it is the female, which has two different sex chromosomes. In some reptiles, females are also the heterogametic sex. This fits well with the findings of a negative PAC-effect found in sand lizards [25].

The hypothesis of progressive telomere erosion in the germline of the monogametic sex is not supported by one report from Madrid on lab mice with a negative PAC-effect [26]. Interestingly, a Spanish research group (including two authors of the previous study) recently published new findings of telomere lengthening in sperm cells of older CD1 outbred mice in a small two-point longitudinal study [27]. The authors found a significant increase of sperm telomere length in four out of eight mice. However, these mice had unusually short sperm telomeres when they were young; actually, their sperm telomeres were shorter than the telomeres in their somatic tissues, which is very odd. In presumably all female mice (the exact number is not mentioned), Ramos-Ibeas et al. reported that telomeres eroded in oocytes at advanced age [27]. Although I strongly welcome the first longitudinal study on germ cell telomere length in mammals, these puzzling results clearly must be verified by others.

## Concluding remarks

The hypothesis of progressive telomere erosion in the germline of the monogametic sex, which is outlined here for the first time, is of course highly speculative, but such alternative interpretations of existing experimental data are, in my mind, essential for future experiments.

Longitudinal studies on germ cell mean telomere length are much needed to be performed by experienced investigators, not by students, preferentially applying the old reliable southern blot techniques. Modern molecular genetic techniques for reliable telomere measurements have to be developed; current PCR methods do not seem to be good enough [28].

Instead of the hundredth cross-sectional study in support of an odd concept, the lengthening of human sperm telomeres during aging (through telomeric extension or through Darwinian selection), the science community must promote longitudinal studies of telomere length in the human germline.

If the human population loses telomeric sequence over generations in a progressive manner [8, 9, 15, 18], we have a problem, because a limited regenerative capacity of somatic tissues (due to short telomeres) will most likely result in an increase in and earlier onset of the so-called age-associated diseases [8, 29].

## Data Availability

Not applicable
